# Supplementary figures and images for: Host adaptation and convergent evolution increases antibiotic resistance without loss of virulence in a major human pathogen
Source: PLoS Pathog. 2019 Mar 15;15(3):e1007218. doi: 10.1371/journal.ppat.1007218 (PMC6436753; doi:10.1371/journal.ppat.1007218)

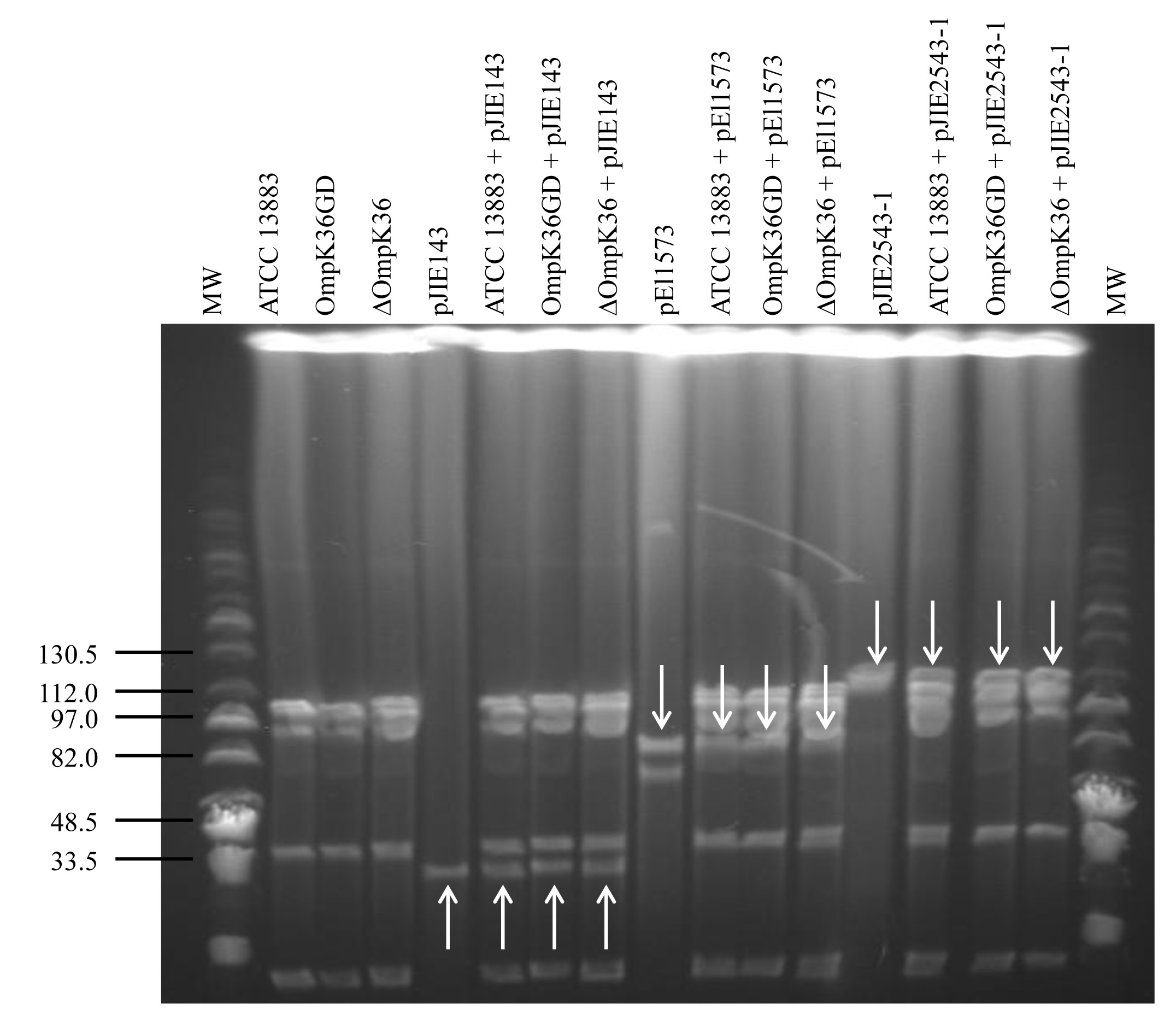

Supplement: S1 Fig — White arrows show the plasmids in original host isolates and transconjugants. MW; Mid-range PFG Marker. (TIF) [file ppat.1007218.s001.tif]

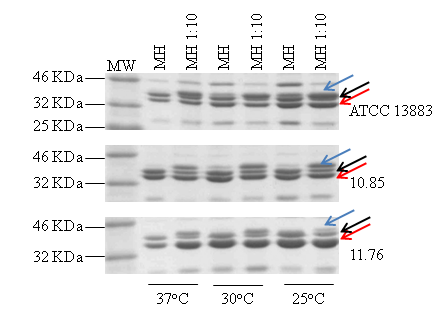

Supplement: S2 Fig — Wild type strains ATCC 13883, 10.85 and 11.76 were cultured under different temperatures (37°C, 30°C and 25°C) and different nutrient concentrations (MH and MH 1:10). Blue arrow, OmpK35. Black arrow, OmpK36. Red arrow, OmpA. (TIF) [file ppat.1007218.s002.tif]

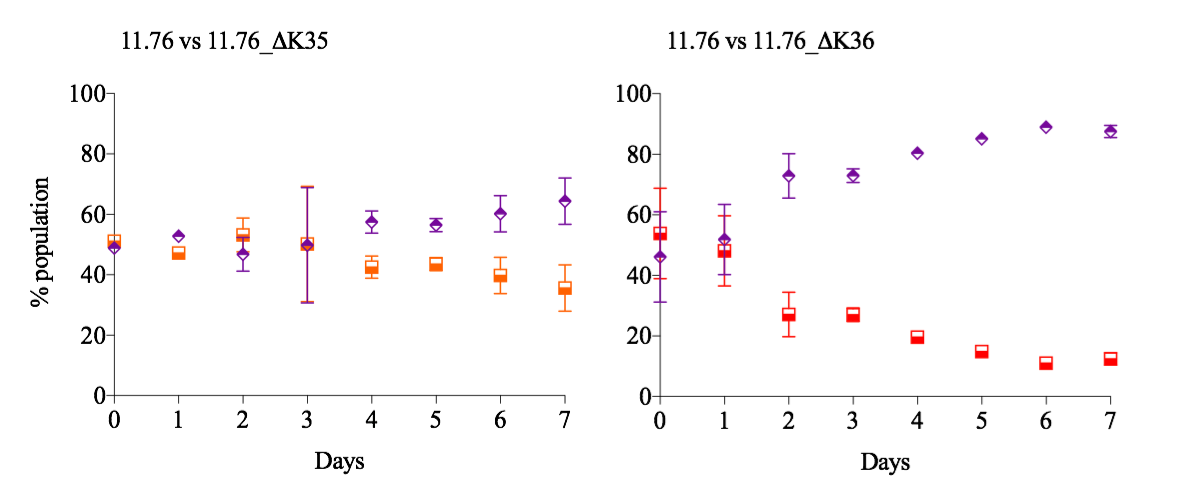

Supplement: S3 Fig — The relative fitness of deletion porin mutants in comparison with parental strain (11.76) was performed by competition experiments in co-cultures and expressed as a percentage of the mutant or wild type cells versus total population at each time point. In vitro growth conditions, MH broth, 37°C. Violet diamond, 11.76 wild type strains. Orange square, ΔOmpK35 mutant. Red square, ΔOmpK36 mutant. (TIFF) [file ppat.1007218.s003.tiff]

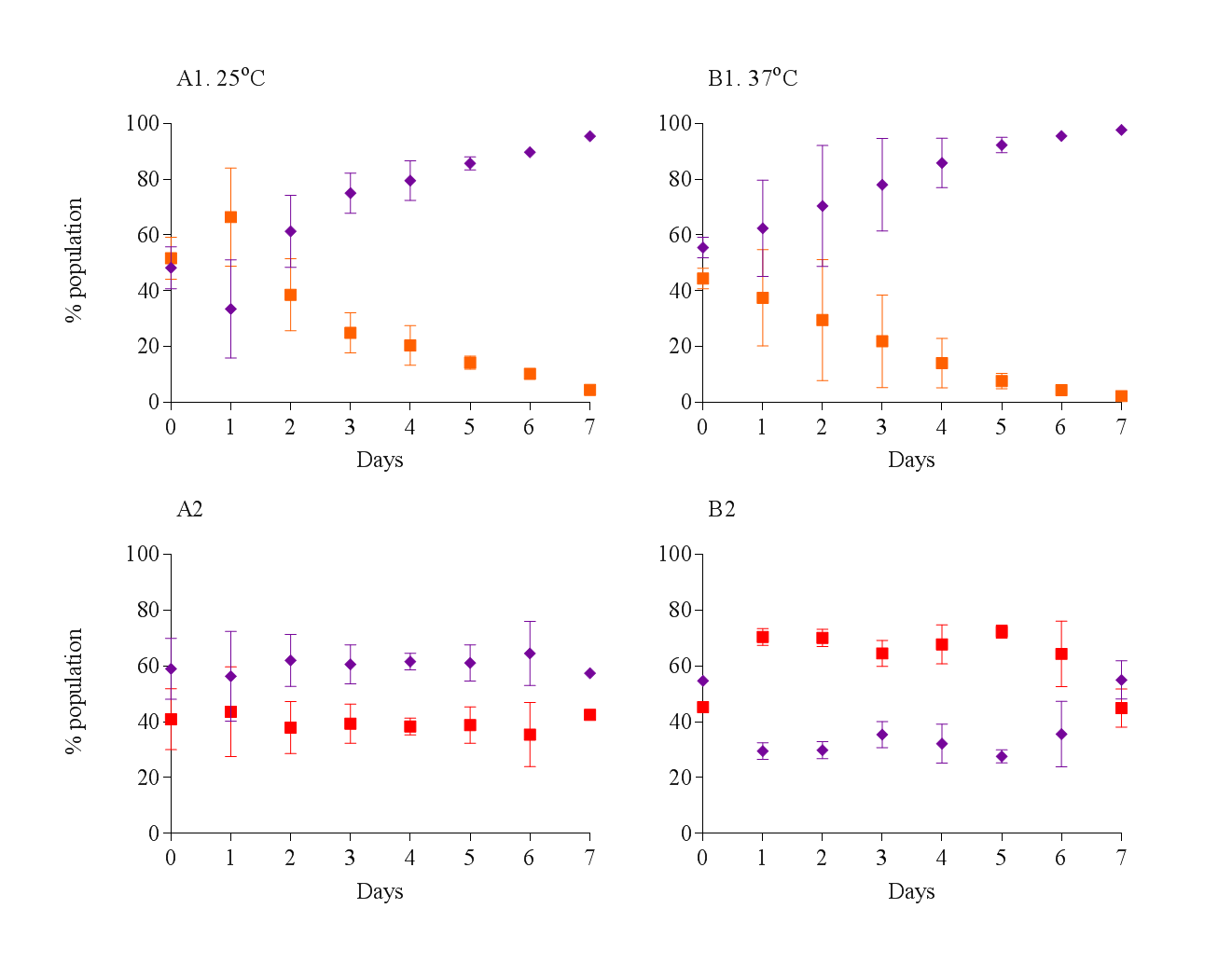

Supplement: S4 Fig — The relative fitness of porin mutants in comparison with parental strain ATCC 13883 was performed by competition experiments in co-cultures and expressed as a percentage of the mutant or wild type cells versus total population at each time point. In vitro growth conditions: A, MH 1:10 broth, 25°C; B, MH 1:10 broth, 37°C. Violet diamond, ATCC 13883. Orange square, ΔOmpK35. Red square, ΔOmpK36. (TIF) [file ppat.1007218.s004.tif]

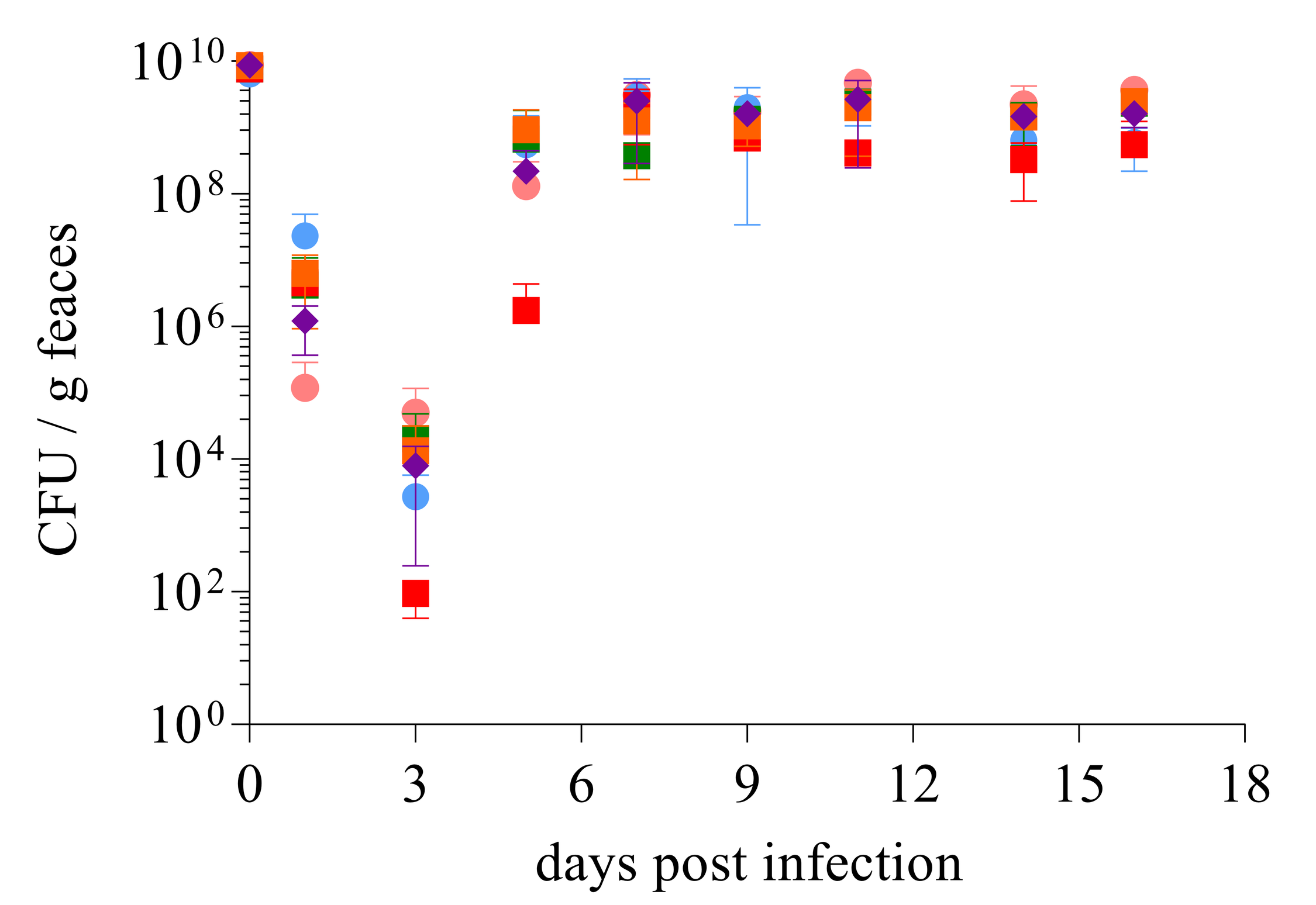

Supplement: S5 Fig — K. pneumoniae intestinal colonization in a mouse model. CFU counts of K. pneumoniae ATCC 13883 and porin mutants from mice faecal sample. Bacterial inoculum at day 0 is 1x10^10^ CFU /mouse. Addition of ampicillin 0.5 g / L in the drinking water on day 4. Violet diamond, ATCC 13883 wild type strains. Orange square, ΔOmpK35 mutant. Red square, ΔOmpK36 mutant. Blue circle, ΔOmpK35ΔOmpK36 mutant. Green square, OmpK36GD mutant. Pink circle, ΔOmpK35OmpK36GD mutant. (TIF) [file ppat.1007218.s005.tif]

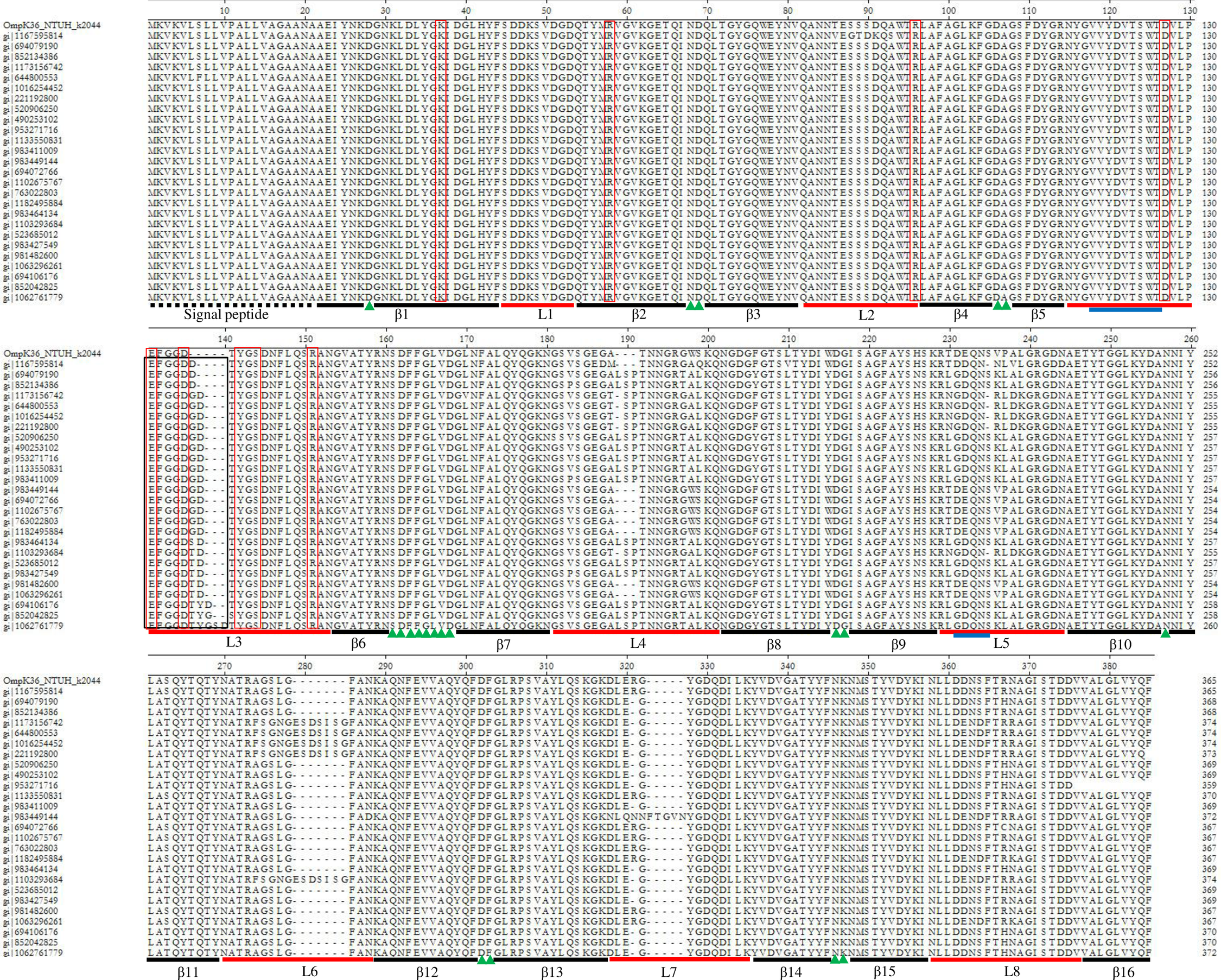

Supplement: S6 Fig — 26 unique sequences with OmpK36 L3 variants from GenBank were compared with OmpK36 of NTUH_K2044. Isolates with wild-type L3 sequence are not included. Dot line, signal peptide. Black line, beta strands. Red line, loops. Blue line, alpha helix. Green squares, turns. OmpK36 secondary structure based on previous studies [82,83]. Red boxes, residues involved in the pore eyelet based on [84]. Black box, L3 variants. (TIF) [file ppat.1007218.s006.tif]

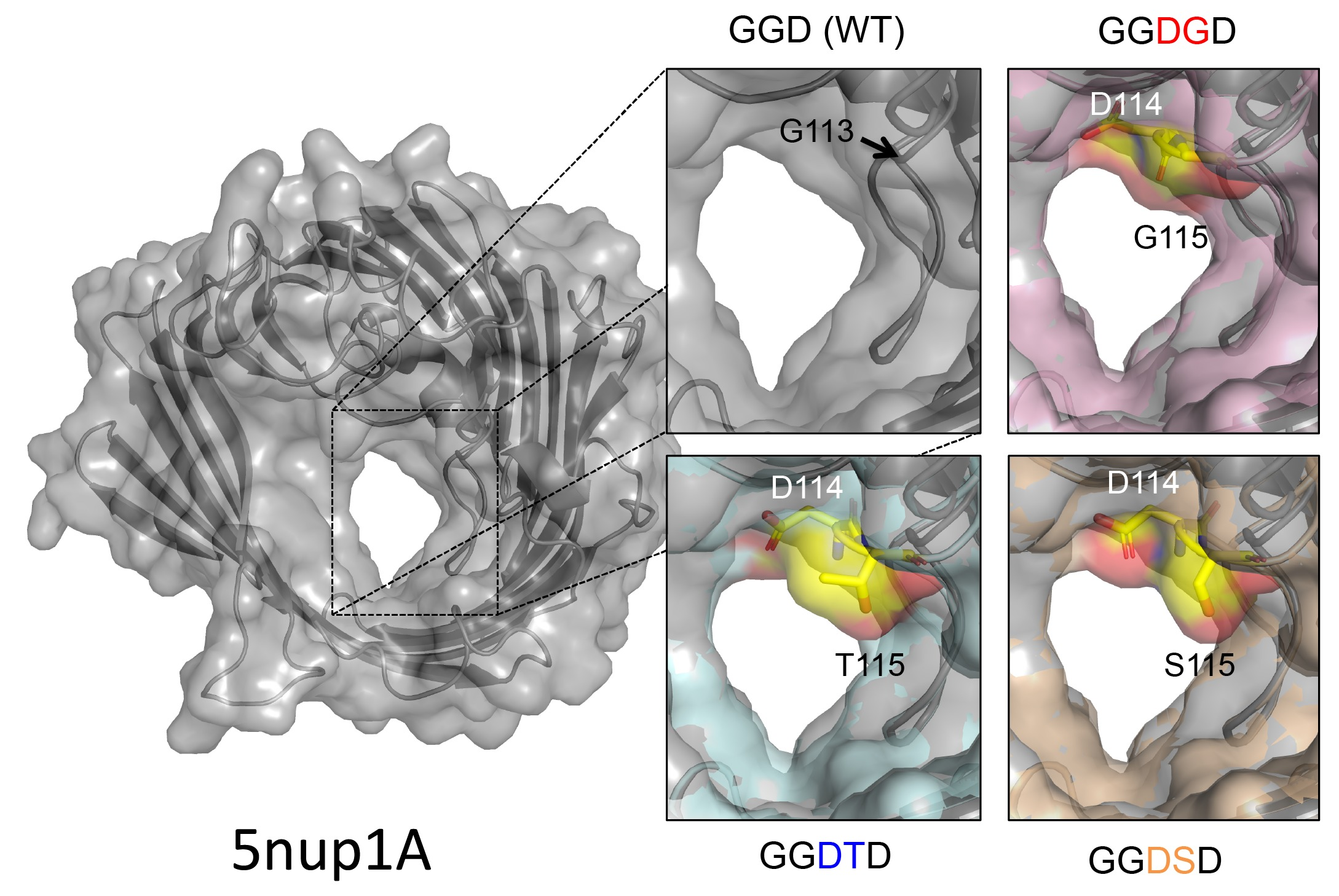

Supplement: S7 Fig — Comparison of the reference OmpK36 structure under PDB accession 5nup1A (WT, wild type) against predicted structural models of mutants harbouring a two amino-acid insertion in loop 3 after G113, namely GGDGD, GGDTD and GGDSD. For each predicted structure, the 2 most protruding amino-acids resulting from the insertion were marked and coloured according to their backbone structure (carbons in yellow, oxygens in red and nitrogens in blue). (TIF) [file ppat.1007218.s007.tif]

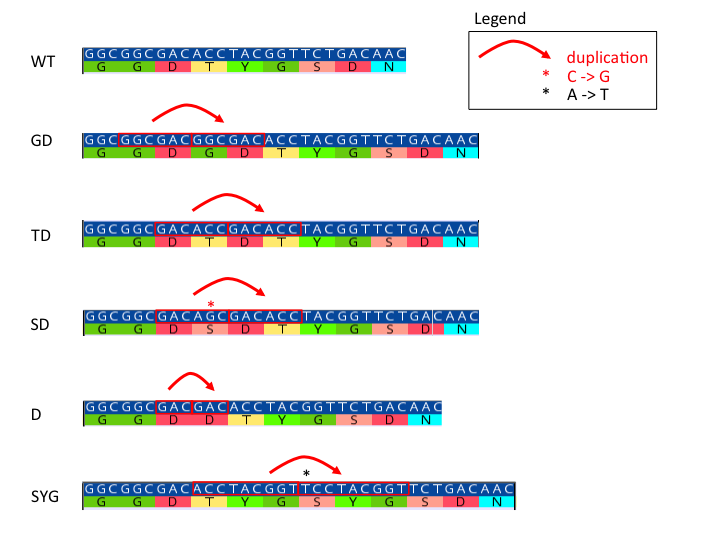

Supplement: S8 Fig — Based on observations of the codon sequences, the extra–SD and–SYG following GGD likely result from a combination of duplication followed by point mutation. (TIF) [file ppat.1007218.s008.tif]

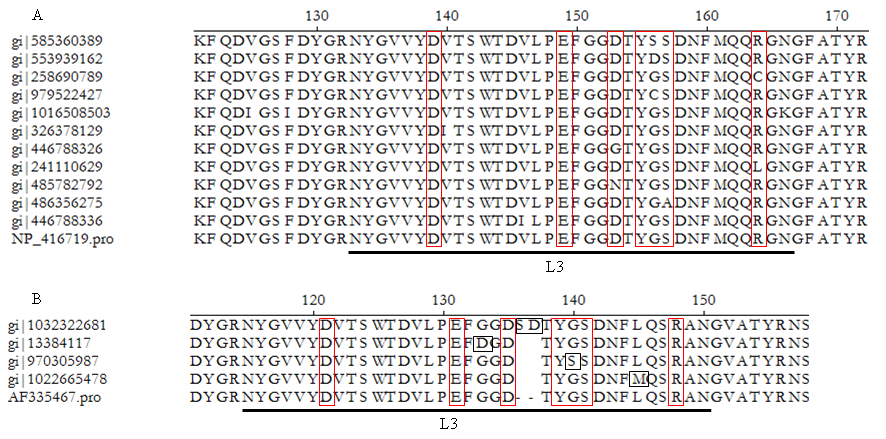

Supplement: S9 Fig — A. Alignment of E. coli OmpC_L3 variants. 11 unique Omp36_L3 variants from GenBank were compared with L3 of OmpC of K-12 MG1655 (NP_416719). Black boxes, residues different from NP_416719. B. Alignment of E. aerogenes Omp36_L3 variants. Four unique Omp36_L3 variants from GenBank were compared with L3 of Omp36 from ATCC 13048 (AF335467). Black boxes, residues different from AF335467. Isolates with wild-type L3 sequence are not included. Black line, loop 3. OmpK36 L3 location based on previous studies. (82, 83). Red boxes, residues involved in the pore eyelet based on (84). (TIF) [file ppat.1007218.s009.tif]

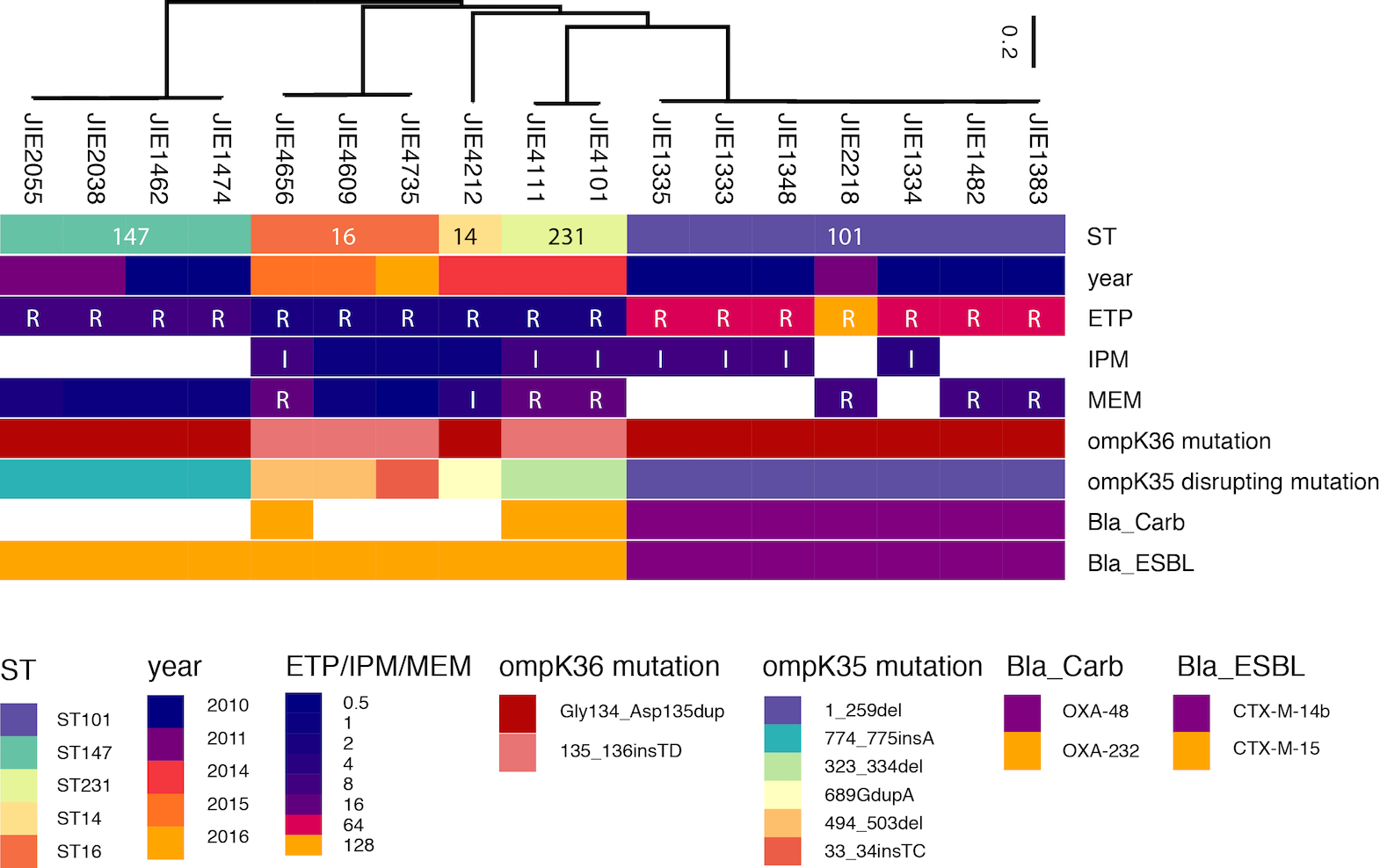

Supplement: S10 Fig — Metadata includes year of isolation; MIC levels for ETP: ertapenem, IMP: imipenem, and MEM: meropenem; ompK36 L3 mutation; ompK35 disrupted mutations (as listed in S7 Table); ST: sequence type; number of predicted resistance genes encoded; carbapenamase gene encoded; ESBL: extended-spectrum beta-lactamase gene encoded. (TIF) [file ppat.1007218.s010.tif]

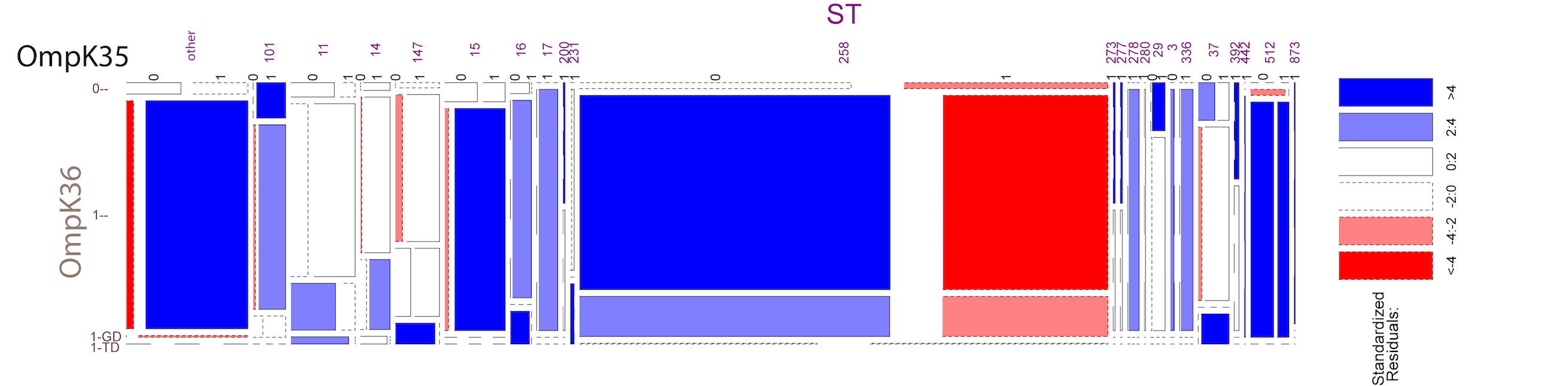

Supplement: S11 Fig — The mosaic plot shows the relationships between 3 variables; ST (in purple) and presence/absence of ompK35 (in black) on the x-axis; and presence/absence and mutations of ompK36 (in grey) on the y-axis. The size of each plot tile is proportional to counts. Plot tiles are colored according to their standardized Pearson residuals, as determined by a log-linear model. Deeper shades of red and blue corresponding to a standardized residual less than -4 or greater than +4, respectively, can be interpreted as combinations observed significantly less or more than expected (under the assumptions that proportions have equal levels). (TIF) [file ppat.1007218.s011.tif]

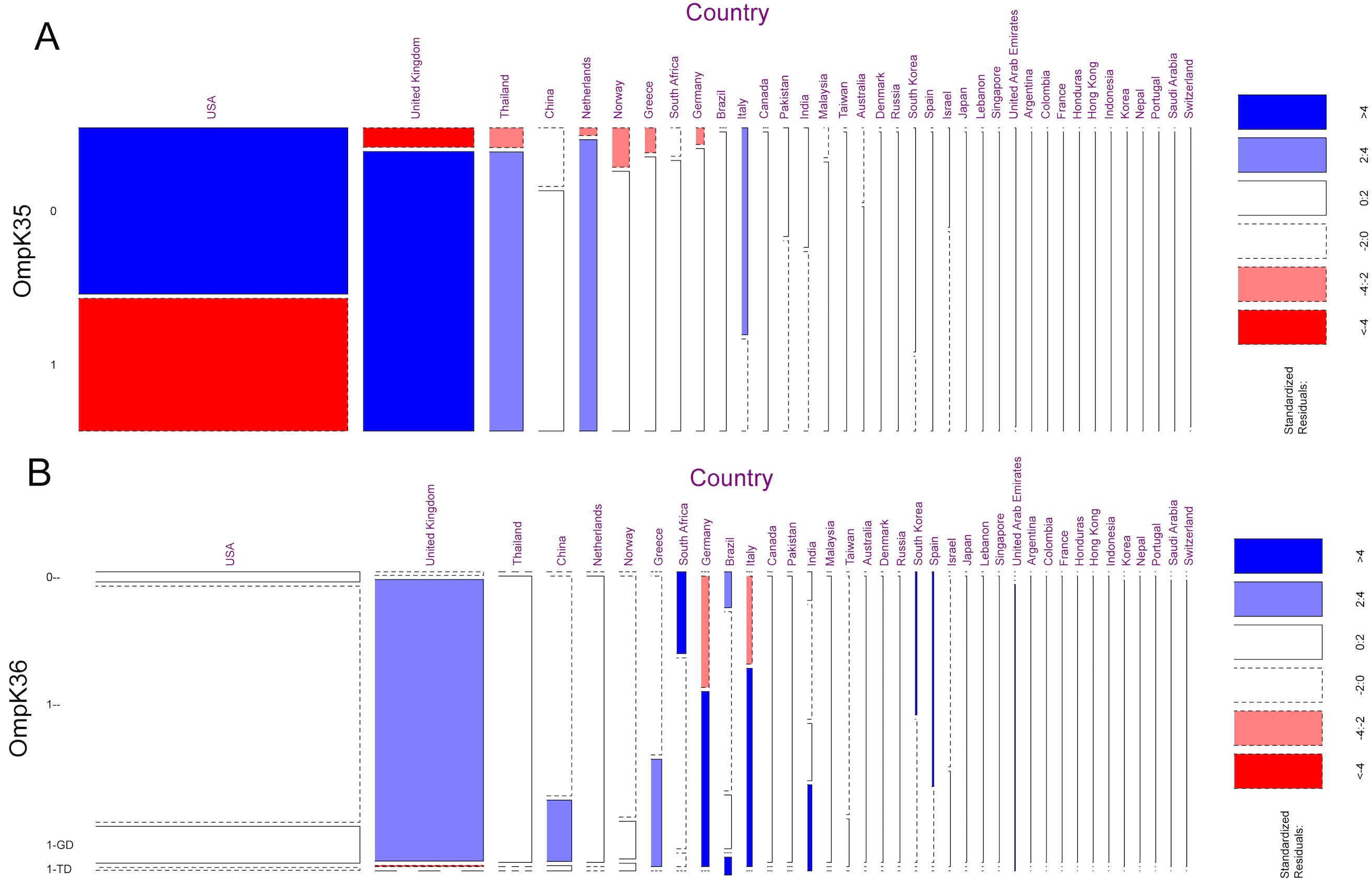

Supplement: S12 Fig — The mosaic plots show the relationships between 2 variables; A) country of isolation on the x-axis and presence/absence of ompK35 on the y-axis; B) country of isolation on the x-axis, and presence/absence and mutations of ompK36 on the y-axis. The size of each plot tile is proportional to counts. Plot tiles are colored according to their standardized Pearson residuals, as determined by a log-linear model. Deeper shades of red and blue corresponding to a standardized residual less than -4 or greater than +4, respectively, can be interpreted as combinations observed significantly less or more than expected (under the assumptions that proportions have equal levels). (TIF) [file ppat.1007218.s012.tif]

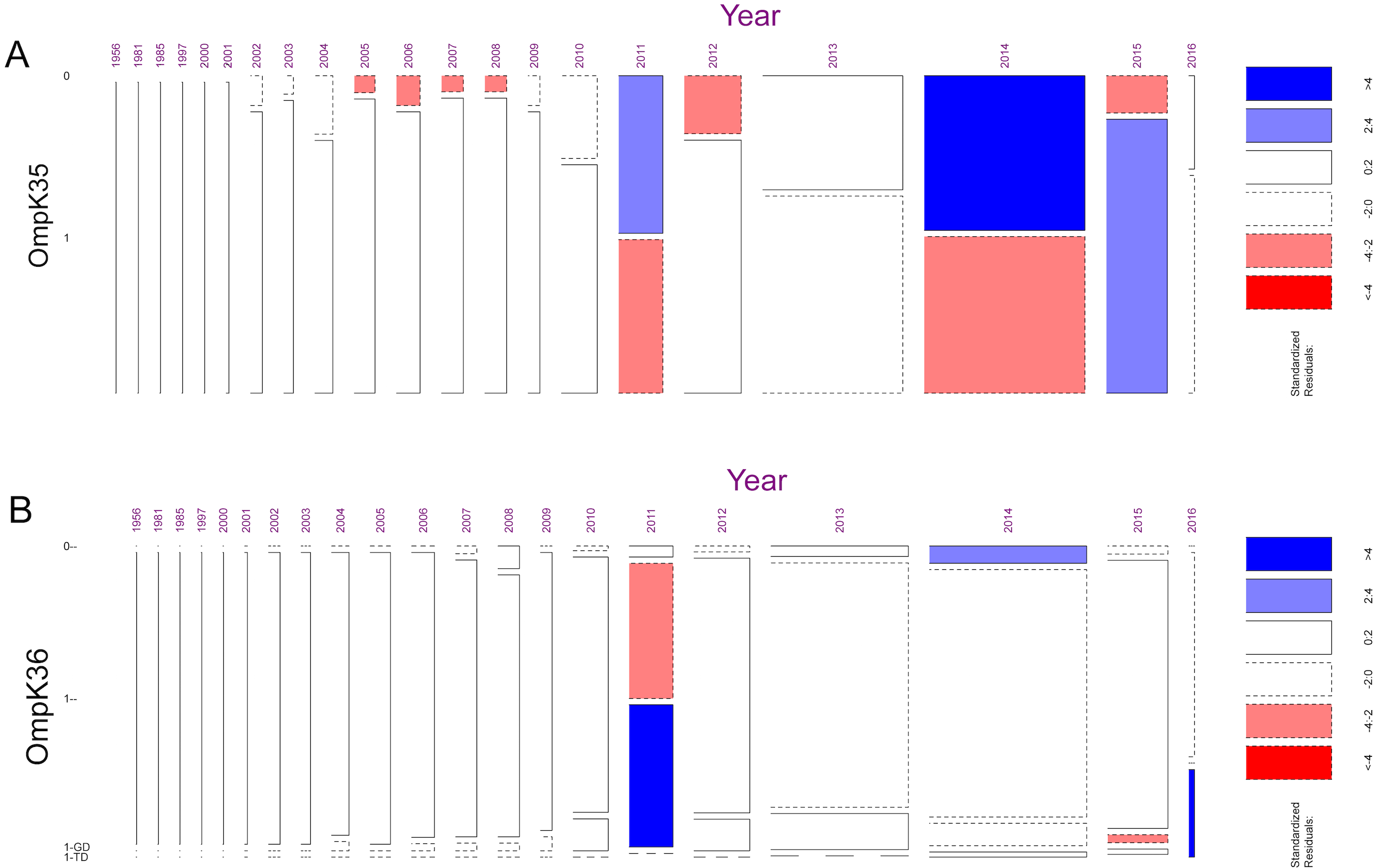

Supplement: S13 Fig — The mosaic plots show the relationships between 2 variables; A) year of isolation on the x-axis and presence/absence of ompK35 on the y-axis; B) year of isolation on the x-axis, and presence/absence and mutations of ompK36 on the y-axis. The size of each plot tile is proportional to counts. Plot tiles are colored according to their standardized Pearson residuals, as determined by a log-linear model. Deeper shades of red and blue corresponding to a standardized residual less than -4 or greater than +4, respectively, can be interpreted as combinations observed significantly less or more than expected (under the assumptions that proportions have equal levels). (TIF) [file ppat.1007218.s013.tif]

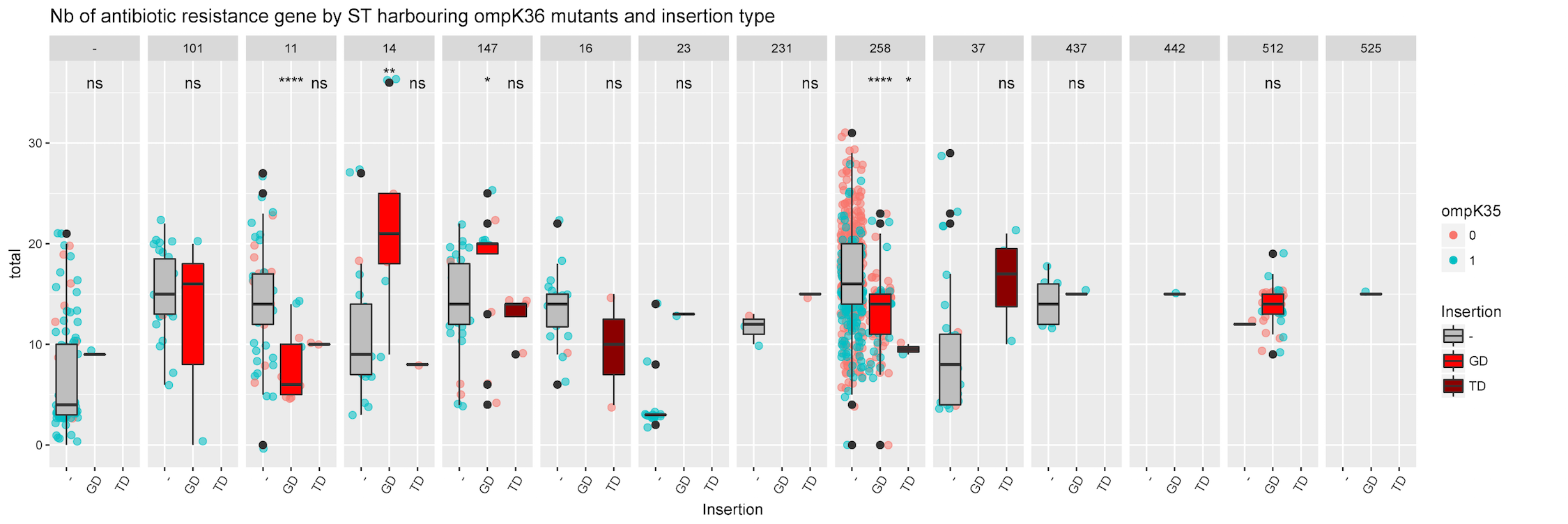

Supplement: S14 Fig — Boxplots were used to display the distribution of resistance genes identified with Abricate within each ST with the following OmpK36 variants, namely isolates with–GD in bright red,–TD in brown, or no insertion (–) in grey. Mean comparison p-values are also shown for each ST (Wilcoxon test, with ‘-’ used as a reference group; ns: p > 0.05; *: p < = 0.05; **: p < = 0.01; ***: p < = 0.001; ****: p < = 0.0001). In addition, the corresponding underlying isolate population is also visualised as individual points, coloured according to OmpK35 type, (1) intact in turquoise or (0) disrupted in coral. (TIF) [file ppat.1007218.s014.tif]
