# Supplementary material for: Host adaptation and convergent evolution increases antibiotic resistance without loss of virulence in a major human pathogen
Source: PLoS Pathog. 2019 Mar 15;15(3):e1007218. doi: 10.1371/journal.ppat.1007218 (PMC6436753; doi:10.1371/journal.ppat.1007218)
Supplement: S1 Table — (DOC) [file ppat.1007218.s015.doc]

| **Table S1** Primers and plasmids used during this work | | | |
| --- | --- | --- | --- |
| **Primer** | **Sequence 5ʼ-3ʼ** | **Target** | **Reference** |
| Imp BE1 | CAYGGTTTGGTGGTTCTTGTAA | *bla*_IMP_ forward | [1] |
| Imp BE2 | CCTTTAACVGCCTGYTCTYMT | *bla*_IMP_ reverse | [1] |
| ISEcp1IR-F | CAATGTGTGAGAAGCAGTCTAAA | *bla*_CTX-M_ forward | [2] |
| Orf477-R | GGTGGCATAATTTTTGAAGT | *bla*_CTX-M_ reverse | [2] |
| KPC-F | ATGTCACTGTATCGCCGTCT | *bla*_KPC_ forward | [3] |
| KPC-R | TTTTCAGAGCCTTACTGCCC | *bla*_KPC_ reverse | [3] |
| **Porin interruption** | | | |
| TetA-F | GAGCCTCTACGCCGACCTCA | *tetA* gene forward | [4] |
| TetA-R | GTAGCCGGAAGTCGCCTTGA | *tetA* gene reverse | [4] |
| AphA3-F | CCCGGGTGACTAACTAGGAGGAATAA | *aphA3* forward | [5] |
| AphA3-R | CCCGGGTCATTATTCCCTCCAGGTACTA | *aphA3* reverse | [5] |
| ompK35X-F | TTGACAGAACTTATTGACGGCAG | Upstream of *ompK35* gene | [5] |
| ompK35X-R | CCGCTTTGGTGTAATCGTTG | Downstream of *ompK35* gene | [5] |
| ompK35-F1 | CGACAAAAAGCGCGAAGGTT | Upstream of *ompK35* gene | [6] |
| ompK35-R2 | CGAAACGTTGTCGCGTATGG | Downstream of *ompK35* gene | [6] |
| ompK36-BE1 | TGCAGCACAATGAAATAGCCGACTG | Upstream of *ompK36* gene | [5] |
| ompK36-BE2 | CAAGAGTATACCAGCGAGGTTAAAC | Downstream of *ompK36* gene | [5] |
| D-K36F1 | GGGAAGAATCGCACGAAATA | Upstream of *ompK36* gene | [7] |
| D-K36R2 | GTGGTGACCGGCTGTTTATC | Downstream of *ompK36* gene | [7] |
| PKM200-F | GGCTCGTATAATGTGTGGA | Upstream of plasmid pKM200 | This study |
| PKM200-R | GATGCCATTCTGCTTATCA | Downstream of plasmid pKM200 | This study |
| SacBF1 | GCTGTACCTCAAGCGAAAGG | Inside *sacB*, pCACtus | This study |
|  |  |  |  |
| **Porin complementation** | |  |  |
| ompK36-QQ1 | GACAAGCTTTAAAAGGCATATAACAAACAG | Upstream of *ompK36* gene | This study |
| ompK36-QQ2 | CTGGGATCCAGCGAGGTTAAACCGG | Downstream of *ompK36* gene | This study |
| **Real time RT-PCR** | | | |
| RT-rpoB-F1 | GCGGTTGGTCGTATGAAGTT | Real time PCR *rpoB* | [8] |
| RT-rpoB-F1 | TGGCGTTGATCATATCCTGA | Real time PCR *rpoB* | [8] |
| RT-K35-F1 | TCCCTGCCCTGCTGGTAG | Real time PCR *ompK35* | [9] |
| RT-K35-R1 | CTGGTGTCGCCATTGGTGG | Real time PCR *ompK35* | [9] |
| RT-K36-F1 | GCGACCAGACCTACATGCGT | Real time PCR *ompK36* | [9] |
| RT-K36-R1 | AGTCGAAAGAGCCCGCGTC | Real time PCR *ompK36* | [9] |
| RT-K37-F1 | CTGGCGACCATGTACTCTGA | Real time PCR *ompK37* | BE |
| RT-K37-R1 | GGCATAGCGGCCGTTATTGT | Real time PCR *ompK37* | BE |
| RT-K26-F1 | CAACCGCGGTAACGATAGAT | Real time PCR *ompK26* | This study |
| RT-K26-R1 | GGTAAAGCCTAAGCCGTTGG | Real time PCR *ompK26* | This study |
| RT-LamB-F1 | ACCGGCGCTCAAAGTAAATA | Real time PCR *lamB* | This study |
| RT-LamB-R1 | GATTCACGGAATAGGCAACG | Real time PCR *lamB* | This study |
| RT-PhoE-F1 | TCGGCACCTCGTTAAGCTAT | Real time PCR *phoE* | BE |
| RT-PhoE-R1 | CATTTTGCGGGTTTCAGAGT | Real time PCR *phoE* | BE |
| **GD duplication** | | | |
| K36GD1 | TTGAAACATCTTGTGGGAACTTT | Upstream of ompK36 | This study |
| ^a^ K36GD2 | CCGTAGGTGTCGCCGTCGCCGCC | ompK36 with Gly/Asp insertion | This study |
| ^a^ K36GD3 | GGCGGCGACGGCGACACCTACGGT | ompK36 with Gly/Asp insertion | This study |
| K36GD4 | AGAGGCGCCTTTTTGTTATG | Downstream of ompK36 | This study |
| ^a^K36GD11 | AATTCGGCGGCGACGGCGAC | Beginning of ompK36 with GD insertion | This study |
| K36GD12 | ATGAAAGTTAAAGTACTGTC | OmpK36 from start codon | [10] |
| ^a^K36GD13 | CGTAGGTGTCGCCGTC | OmpK36 with GD insertion | This study |
| **Plasmids** | **General characteristics** | | **Reference or source** |
| pCACtus | temperature-sensitive, sucrose-sensitive (*sac*B), chloramphenicol-resistant (*cat*) | | CC^b^ |
| pJIAF-7 | ATCC 13883 *ompK35:tetA* cloned in pCACtus | | This study |
| pJIAF-8 | ATCC 13883 *ompK36:aphA-3* cloned in pCACtus | | This study |
| pJIAF-9 | 10.85 *ompK35:tetA* cloned in pCACtus | | This study |
| pJIAF-10 | 10.85 *ompK36:aphA-3* cloned in pCACtus | | This study |
| pJIAF-11 | 11.76 *ompK35:tetA* cloned in pCACtus | | This study |
| pJIAF-12 | 11.76 *ompK36:aphA-3* cloned in pCACtus | | This study |
| pJIAF-13 | ATCC 13883 *ompK35:tetA* cloned in pCACtus | |  |
| pJIAF-14 | ATCC 13883 *ompK36:aphA-3* cloned in pCACtus | |  |
| pJIAF-15 | 10.85 *ompK35:tetA* cloned in pCACtus | |  |
| pJIAF-16 | 10.85 *ompK36:aphA-3* cloned in pCACtus | |  |
| pJIAF-17 | 11.76 *ompK35:tetA* cloned in pCACtus | |  |
| pJIAF-18 | 11.76 *ompK36:aphA-3* cloned in pCACtus | |  |
| pKM200 | λ Red- mediated recombinogenic plasmid (Ptac-gam-red), chloramphenicol resistant | | Addgene, USA [11, 12] |
| pGEM-T | Ampicillin resistant cloning vector | | Promega |
| pJIAF-1 | ATCC 13883 *ompK35:tetA* cloned in pGEM-T | | This study |
| pJIAF-2 | ATCC 13883 *ompK36:aphA-3* cloned in pGEM-T | | This study |
| pJIAF-3 | 10.85 *ompK35:tetA* cloned in pGEM-T | | This study |
| pJIAF-4 | 10.85 *ompK36:aphA-3* cloned in pGEM-T | | This study |
| pJIAF-5 | 11.76 *ompK35:tetA* cloned in pGEM-T | | This study |
| pJIAF-6 | 11.76 *ompK36:aphA-3* cloned in pGEM-T | | This study |
| pJIE143 | Plasmid carrying *bla*_CTX-M-15_ | | [13] |
| pEl1573 | Plasmid carrying *bla*_IMP-4_ | | [14] |
| pJIE2543-1 | Plasmid carrying *bla*_KPC-2_ | | [6] |
| pACYC-184 | Chloramphenicol, tetracycline resistant expression vector | | [15] |
| pJIQQ-1 | ATCC 13883 *ompK36* cloned in pACYC-184 | | This study |
| pJIQQ-2 | ATCC 13883 *ompK36GD* cloned in pACYC-184 | | This study |
| ^a^GlyAsp duplication underlined  ^b^pCACtus-mob was obtained from Mr. C. Cursaro (CC, Dept. Microbiol. Immunol. University of Adelaide, Australia).  BE: primers designed by Bjorn A. Espedido | | | |

1. Espedido BA, Partridge SR, Iredell JR. *bla*_IMP-4_ in different genetic contexts in *Enterobacteriaceae* isolates from Australia. Antimicrobial agents and chemotherapy. 2008;52(8):2984-7. Epub 2008/05/21. doi: 10.1128/AAC.01634-07. PubMed PMID: 18490506; PubMed Central PMCID: PMC2493119.

2. Zong Z, Partridge SR, Thomas L, Iredell JR. Dominance of *bla*_CTX-M_ within an Australian extended-spectrum β-lactamase gene pool. Antimicrobial agents and chemotherapy. 2008;52(11):4198-202. doi: 10.1128/AAC.00107-08. PubMed PMID: 18725449; PubMed Central PMCID: PMC2573124.

3. Bradford PA, Bratu S, Urban C, Visalli M, Mariano N, Landman D, et al. Emergence of carbapenem-resistant *Klebsiella* species possessing the class A carbapenem-hydrolyzing KPC-2 and inhibitor-resistant TEM-30 β-lactamases in New York City. Clinical infectious diseases : an official publication of the Infectious Diseases Society of America. 2004;39(1):55-60. doi: 10.1086/421495. PubMed PMID: 15206053.

4. Kamruzzaman M, Shoma S, Thomas CM, Partridge SR, Iredell JR. Plasmid interference for curing antibiotic resistance plasmids in vivo. PloS one. 2017;12(2):e0172913. Epub 2017/03/01. doi: 10.1371/journal.pone.0172913. PubMed PMID: 28245276; PubMed Central PMCID: PMC5330492.

5. Jiang X, Espedido BA, Partridge SR, Thomas LC, Wang F, Iredell JR. Paradoxical effect of *Klebsiella pneumoniae* OmpK36 porin deficiency. Pathology. 2009;41(4):388-92. doi: 10.1080/00313020902908854. PubMed PMID: 19404854.

6. Partridge SR, Ginn AN, Wiklendt AM, Ellem J, Wong JS, Ingram P, et al. Emergence of *bla*_KPC_ carbapenemase genes in Australia. Int J Antimicrob Agents. 2015;45(2):130-6. Epub 2014/12/04. doi: 10.1016/j.ijantimicag.2014.10.006. PubMed PMID: 25465526.

7. Agyekum A, Fajardo-Lubian A, Ai X, Ginn AN, Zong Z, Guo X, et al. Predictability of phenotype in relation to common β-lactam resistance mechanisms in *Escherichia coli* and *Klebsiella pneumoniae*. Journal of clinical microbiology. 2016;54(5):1243-50. Epub 2016/02/26. doi: 10.1128/jcm.02153-15. PubMed PMID: 26912748; PubMed Central PMCID: PMC4844708.

8. Srinivasan VB, Venkataramaiah M, Mondal A, Vaidyanathan V, Govil T, Rajamohan G. Functional characterization of a novel outer membrane porin KpnO, regulated by PhoBR two-component system in Klebsiella pneumoniae NTUH-K2044. PloS one. 2012;7(7):e41505. Epub 2012/08/01. doi: 10.1371/journal.pone.0041505. PubMed PMID: 22848515; PubMed Central PMCID: PMC3405095.

9. Doumith M, Ellington MJ, Livermore DM, Woodford N. Molecular mechanisms disrupting porin expression in ertapenem-resistant *Klebsiella* and *Enterobacter* spp. clinical isolates from the UK. The Journal of antimicrobial chemotherapy. 2009;63(4):659-67. doi: 10.1093/jac/dkp029. PubMed PMID: 19233898.

10. Garcia-Fernandez A, Miriagou V, Papagiannitsis CC, Giordano A, Venditti M, Mancini C, et al. An ertapenem-resistant extended-spectrum-beta-lactamase-producing Klebsiella pneumoniae clone carries a novel OmpK36 porin variant. Antimicrobial agents and chemotherapy. 2010;54(10):4178-84. Epub 2010/07/28. doi: 10.1128/AAC.01301-09. PubMed PMID: 20660683; PubMed Central PMCID: PMC2944588.

11. Murphy KC, Campellone KG. Lambda Red-mediated recombinogenic engineering of enterohemorrhagic and enteropathogenic *E. coli*. BMC Mol Biol. 2003;4:11. doi: 10.1186/1471-2199-4-11. PubMed PMID: 14672541; PubMed Central PMCID: PMCPMC317293.

12. Datsenko KA, Wanner BL. One-step inactivation of chromosomal genes in *Escherichia coli* K-12 using PCR products. Proc Natl Acad Sci U S A. 2000;97(12):6640-5. doi: 10.1073/pnas.120163297. PubMed PMID: 10829079; PubMed Central PMCID: PMCPMC18686.

13. Partridge SR, Ellem JA, Tetu SG, Zong Z, Paulsen IT, Iredell JR. Complete sequence of pJIE143, a pir-type plasmid carrying IS*Ecp1*-*bla*_CTX-M-15_ from an *Escherichia coli* ST131 isolate. Antimicrobial agents and chemotherapy. 2011;55(12):5933-5. Epub 2011/09/14. doi: 10.1128/AAC.00639-11. PubMed PMID: 21911569; PubMed Central PMCID: PMC3232798.

14. Partridge SR, Ginn AN, Paulsen IT, Iredell JR. pEl1573 carrying *bla*_IMP-4_, from Sydney, Australia, is closely related to other IncL/M plasmids. Antimicrobial agents and chemotherapy. 2012;56(11):6029-32. Epub 2012/08/29. doi: 10.1128/AAC.01189-12. PubMed PMID: 22926566; PubMed Central PMCID: PMC3486572.

15. Chang AC, Cohen SN. Construction and characterization of amplifiable multicopy DNA cloning vehicles derived from the P15A cryptic miniplasmid. J Bacteriol. 1978;134(3):1141-56. Epub 1978/06/01. PubMed PMID: 149110; PubMed Central PMCID: PMCPMC222365.
