# Supplementary material for: Host adaptation and convergent evolution increases antibiotic resistance without loss of virulence in a major human pathogen
Source: PLoS Pathog. 2019 Mar 15;15(3):e1007218. doi: 10.1371/journal.ppat.1007218 (PMC6436753; doi:10.1371/journal.ppat.1007218)
Supplement: S4 Table — (DOC) [file ppat.1007218.s018.doc]

| **Table S4** Antibiotic MICs against *K. pneumoniae* sensitive strains and porin mutants | | | | | | | | | |  |
| --- | --- | --- | --- | --- | --- | --- | --- | --- | --- | --- |
|  | **Antibiotics, MIC (mg/L)^a^** | | | | | | | | |  |
| Strain | **ETP** | **MEM** | **CXM** | **CAZ** | **FOX^b^** | **CEF^b^** | **CFZ^b^** | **CRO** | **CTX** | **AMP** |
| ATCC 13883 | 0.015 | 0.03 | 4-8 | 0.5 | 8 | 8 | 2 | 0.125 | 0.06 | 256 |
| ΔK35 | 0.03 | 0.06 | 8 | 1 | 16 | 8-16 | 4 | 0.25 | 0.125 | 256 |
| ΔK36 | **0.0625** | 0.06 | 16 | 1 | 16-32 | **32** | **8-16** | 0.25-0.5 | **0.25** | 256-512 |
| ΔK35ΔK36 | **1** | **0.125-0.25** | **32** | 1 | **64** | **64** | **64** | **0.5** | **0.5** | 256-512 |
| K36GD | 0.03-0.06 | 0.03-0.06 | 16 | 1 | 16-32 | 16-32 | 4-8 | 0.25 | **0.25** | 256 |
| ΔK35K36GD | **0.25** | 0.06 | 16 | 1 | **64** | **32-64** | **32-64** | **0.5** | **0.5** | 256-512 |
| 10-85 | 0.015 | 0.03 | 2 | 0.25 | 2 | 2 | 1-2 | 0.06 | 0.03 |  |
| ΔK35 | 0.015 | 0.03 | 4 | **1** | **8** | 4 | 2 | 0.125 | 0.06 |  |
| ΔK36 | 0.015 | 0.03 | 4-8 | 0.25 | **8** | **16** | 4 | 0.125 | **0.125** |  |
| ΔK35ΔK36 | **1** | **0.125** | **16** | **1** | **32** | **32** | **16-32** | **0.25** | **0.25** |  |
| K36GD | 0.03 | 0.03 | **8** | 0.5 | **8** | **8-16** | **4** | **0.25** | **0.25** |  |
| ΔK35K36GD | **0.25** | 0.06 | **16** | **1** | **32** | **32** | **16** | **0.25** | **0.5** |  |
| 11-76 | 0.015 | 0.03 | 4 | 0.5 | 4 | 4 | 2 | 0.06-0.125 | 0.06 |  |
| ΔK35 | 0.015 | 0.03 | 8 | 1 | 8 | 8 | 4 | 0.125 | 0.125 |  |
| ΔK36 | 0.015 | 0.03 | 8 | 0.25 | 8-16 | **16** | 2 | 0.125 | 0.125 |  |
| ΔK35ΔK36 | **1** | **0.125** | **16** | 1 | **32** | **32** | **32** | **0.5** | **0.25** |  |
| K36GD | 0.015 | 0.03 | 8 | 0.5 | 8 | **16** | 4-8 | 0.125 | **0.25** |  |
| ΔK35K36GD | **0.25** | 0.06 | **16** | 1 | **32** | **32** | **32** | 0.25 | **0.5** |  |

MIC. Minimal Inhibitory Concentration. ETP, Ertapenem (S ≤ 0.5, R > 1). MEM, Meropenem (S ≤ 2, R > 8). CXM, Cefuroxime (S ≤ 8, R > 8). CAZ, Ceftazidime (S ≤ 1, R > 4). FOX, Cefoxitin (S ≤ 8, R ≥ 32). CEF, Cephalothin (S ≤ 8, R ≥ 32). CFZ, Cefazolin (S ≤ 2, R ≥ 8). CRO, Ceftriaxone (S ≤ 1, R > 2). CTX, Cefotaxime (S ≤ 1, R > 2). AMP, Ampicillin (S ≤ 8, R > 8).

^a^Boldface numbers indicate at least 4-fold increase between the MICs of the parental strain (*K. pneumoniae* ATCC 13883, 10.85 or 11.76) and the porin mutants. The underlined numbers were intermediate or resistant based on EUCAST breakpoints .

^b^CLSI susceptibility breakpoints . Cefoxitin, cephalothin and cefazolin breakpoints were determined by CLSI guidelines because the breakpoints are not available from EUCAST.
