# Supplementary material for: Host adaptation and convergent evolution increases antibiotic resistance without loss of virulence in a major human pathogen
Source: PLoS Pathog. 2019 Mar 15;15(3):e1007218. doi: 10.1371/journal.ppat.1007218 (PMC6436753; doi:10.1371/journal.ppat.1007218)
Supplement: S5 Table — (DOC) [file ppat.1007218.s019.doc]

**Table S5** Antibiotic MICs against *K. pneumoniae* porin mutants and complemented strains

|  | **MIC (mg/L)^a^** | | | | | |
| --- | --- | --- | --- | --- | --- | --- |
| Strain | **ETP** | **IPM** | **MEM** | **CEF^b^** | **CFZ^b^** | **FOX^b^** |
| 10.85 ΔOmpK35 ΔOmpK36 + pACYC |  |  |  | 32 | 32 | 32 - 64 |
| 10.85 ΔOmpK35 ΔOmpK36 + pACYC_OmpK36 |  |  |  | **1 - 2** | **1 - 2** | **2 -4** |
| 10.85 ΔOmpK35 ΔOmpK36 + pACYC_OmpK36GD |  |  |  | 32 - 64 | 32 - 64 | 32 - 64 |
| JIE2771 + pACYC | 512 | 128 - 256 | 128 |  |  |  |
| JIE2771 + pACYC_OmpK36 | 256 -512 | 32 - 128 | **32 - 64** |  |  |  |
| JIE2771 + pACYC_OmpK36GD | 256 -512 | 64 - 128 | 64 - 128 |  |  |  |

MIC. Minimal Inhibitory Concentration. ETP, Ertapenem (S ≤ 0.5, R > 1). IPM, Meropenem (S ≤ 2, R > 8). MEM, Meropenem (S ≤ 2, R > 8). CEF, Cephalothin (S ≤ 8, R ≥ 32). CFZ, Cefazolin (S ≤ 2, R ≥ 8). FOX, Cefoxitin (S ≤ 8, R ≥ 32). MIC values were tested by E-test.

^a^Boldface numbers indicate at least 2-fold decrease between the MICs of strain harbouring empty plasmid (*K. pneumoniae* 10.85 or JIE2771) and the porin mutants complemented. The underlined numbers were intermediate or resistant based on EUCAST breakpoints .

^b^CLSI susceptibility breakpoints . Cefoxitin, cephalothin and cefazolin breakpoints were determined by CLSI guidelines because the breakpoints are not available from EUCAST.
