# Supplementary material for: Host adaptation and convergent evolution increases antibiotic resistance without loss of virulence in a major human pathogen
Source: PLoS Pathog. 2019 Mar 15;15(3):e1007218. doi: 10.1371/journal.ppat.1007218 (PMC6436753; doi:10.1371/journal.ppat.1007218)
Supplement: S6 Table — The expression of rpoD was used to normalize the results. The levels of expression of each mutant are shown relative to the wild type strain ATCC 13883 or 10.85. (DOC) [file ppat.1007218.s020.doc]

**Table S6.** Real-time RT-PCR in *K. pneumoniae* ATCC 13883 and 10.85 porin mutants.

|  |  |  | **Porin mutants** |  |  |
| --- | --- | --- | --- | --- | --- |
| **Genes** | **ΔK35** | **ΔK36** | **ΔK35ΔK36** | **K36GD** | **ΔK35K36GD** |
| **ATCC13883** |  |  |  |  |  |
| *ompK26* | 0.95±1.10 | 1.69±0.04 | 1.29±0.23 | 1.11±0.44 | 1.23±0.11 |
| *ompK35* | NA | **5.58±0.21^*^** | NA | 0.65±0.17 | NA |
| *ompK36* | 0.57±0.22 | NA | NA | 1.00±0.26 | 0.86±0.09 |
| *ompK37* | 0.56±0.12 | 0.63±0.17 | 1.33±0.21 | 1.28±0.29 | 0.88±0.18 |
| *phoE* | 0.78±0.60 | 1.48±0.07 | **2.05±0.09^*^** | 1.20±0.58 | 0.98±0.04 |
| *lamB* | 1.05±0.19 | 0.80±0.29 | 1.70±0.78 | 1.18±0.06 | 1.06±0.07 |
| **10.85** |  |  |  |  |  |
| *ompK26* | 1.06±0.28 | 1.06±0.07 | 1.67±0.05 | 1.18±0.21 | 1.094±0.18 |
| *ompK35* | NA | **3.71±0.08^*^** | NA | 0.95±0.08 | NA |
| *ompK36* | 0.77±0.07 | NA | NA | 1.10±0.08 | 1.17±0.10 |
| *ompK37* | 0.97±0.23 | 1.37±0.03 | **2.44±0.10^*^** | 1.06±0.19 | 1.30±0.19 |
| *phoE* | 1.22±0.07 | **1.92±0.09^*^** | **4.47±0.17^*^** | 1.43±0.37 | 0.85±0.17 |
| *lamB* | 1.75±0.04 | **2.33±0.05^*^** | **3.23±0.07^*^** | 1.01±0.37 | 1.34±0.14 |

The expression of *rpoD* was used to normalize the results. The levels of expression of each mutant are shown relative to the wild type strain ATCC 13883 or 10.85.

^*^ The differences between the wild type and the porin mutants were statistically significant (P < 0.05).
