# Supplementary material for: Host adaptation and convergent evolution increases antibiotic resistance without loss of virulence in a major human pathogen
Source: PLoS Pathog. 2019 Mar 15;15(3):e1007218. doi: 10.1371/journal.ppat.1007218 (PMC6436753; doi:10.1371/journal.ppat.1007218)
Supplement: S7 Table — (DOC) [file ppat.1007218.s021.doc]

| **Table S7A.** Relative growth rate and doubling time of *K. pneumoniae* and porin mutants | | |
| --- | --- | --- |
| Strain | relative μ | Td (minutes) |
| ATCC 13883 | 1 | 32.5±1.5 |
| ΔK35 | 0.99±0.01 | 32.9±1.6 |
| ΔK36 | 1.02±0.03 | 32.0±1.7 |
| K36GD | 1.04±0.06 | 31.9±2.5 |
| ΔK35ΔK36 | **0.91±0.04** | **35.9±1.6^*^** |
| ΔK35K36GD | 0.99±0.06 | 33.1±1.2 |
| 10.85 | 1 | 28.7±1.1 |
| ΔK35 | 0.965±0.002 | 29.5±1.2 |
| ΔK36 | 1.013±0.008 | 28.4±0.9 |
| K36GD | 1.024±0.022 | 28.1±0.7 |
| ΔK35ΔK36 | **0.897±0.009** | **32.0±1.5^*^** |
| ΔK35K36GD | 0.991±0.020 | 29.0±1.2 |
| 11.76 | 1 | 27.8±0.1 |
| ΔK35 | 1.02±0.01 | 27.2±0.2 |
| ΔK36 | 0.99±0.01 | 27.9±0.2 |
| K36GD | 1.00±0.01 | 27.7±0.2 |
| ΔK35ΔK36 | **0.90±0.04** | **30.9±1.5^*^** |
| ΔK35K36GD | 0.99±0.02 | 28.1±0.6 |

μ. Growth rate. Td. Doubling time. Growth rates and doubling times were calculated on OD_600_ values between 0.02-0.09 which was considered to be exponential phase . The relative growth rate was calculated by dividing the generation time of each mutant by the generation time of the parental strain (*K. pneumoniae* ATCC 13883, 10.85 or 11.76).

^*^ The differences between the wild type and the porin mutants were statistically significant (P < 0.05).

| **Table S7B.** Relative growth rate and doubling time of *K. pneumoniae* porin mutants  complemented | | |
| --- | --- | --- |
| Strain | relative μ | Td (minutes) |
| 10.85ΔK35ΔK36 + pACYC-184 | 1 | 33.8±0.6 |
| 10.85ΔK35ΔK36 + pACYC-K36 | **1.141±0.006** | **29.5±0.8^*^** |
| 10.85ΔK35ΔK36 + pACYC-K36GD | **1.117±0.004** | **30.4±0.6^*^** |
| JIE2771 + pACYC-184 | 1 | 34.2±0.6 |
| JIE2771 + pACYC-K36 | **1.122±0.004** | **30.5±0.6^*^** |
| JIE2771 + pACYC-K36GD | **1.098±0.005** | **31.4±0.6^*^** |

μ. Growth rate. Td. Doubling time. Growth rates and doubling times were calculated on OD_600_ values between 0.02-0.09 which was considered to be exponential phase . The relative growth rate was calculated by dividing the generation time of each mutant expressing *in trans ompK36* or *ompK36*GD by the generation time of the double deletion OmpK35/OmpK36 strain harbouring pACYC-184 empty vector (*K. pneumoniae* 10.85 or JIE2771).

^*^ The differences between the wild type and the porin mutants were statistically significant (P < 0.05).

1. Knopp M, Andersson DI. Amelioration of the Fitness Costs of Antibiotic Resistance Due To Reduced Outer Membrane Permeability by Upregulation of Alternative Porins. Molecular biology and evolution. 2015;32(12):3252-63. Epub 2015/09/12. doi: 10.1093/molbev/msv195. PubMed PMID: 26358402.
